# A Monitoring System for Vegetable Greenhouses based on a Wireless Sensor Network

**DOI:** 10.3390/s101008963

**Published:** 2010-10-08

**Authors:** Xiu-hong Li, Xiao Cheng, Ke Yan, Peng Gong

**Affiliations:** 1 College of Global Change and Earth System Science, Beijing Normal University, Xinjiekouwai Street No.19, Beijing, 100875, China; 2 State Key Laboratory of Remote Sensing Science, Jointly Sponsored by the Institute of Remote Sensing Applications of Chinese Academy of Sciences and Beijing Normal University, P.O.BOX 9718, Beijing, 100101, China; E-Mails: irsa2008@sina.com (K.Y.); gong@irsa.ac.cn (P.G.)

**Keywords:** wireless sensor network, embedded operating system, environment monitoring, data center, base station

## Abstract

A wireless sensor network-based automatic monitoring system is designed for monitoring the life conditions of greenhouse vegetatables. The complete system architecture includes a group of sensor nodes, a base station, and an internet data center. For the design of wireless sensor node, the JN5139 micro-processor is adopted as the core component and the Zigbee protocol is used for wireless communication between nodes. With an ARM7 microprocessor and embedded ZKOS operating system, a proprietary gateway node is developed to achieve data influx, screen display, system configuration and GPRS based remote data forwarding. Through a Client/Server mode the management software for remote data center achieves real-time data distribution and time-series analysis. Besides, a GSM-short-message-based interface is developed for sending real-time environmental measurements, and for alarming when a measurement is beyond some pre-defined threshold. The whole system has been tested for over one year and satisfactory results have been observed, which indicate that this system is very useful for greenhouse environment monitoring.

## 1. Introduction

Againts the background of global informatization and digitization, traditional agriculture is gradually turning into digital agriculture. Greenhouse cultivation is the major method of vegetable production in many areas of China. Although some modern greenhouses are emerging, traditional greenhouses account for the majority of those used in China. Since most greenhouses are poorly equipped with backwards facilities, farmers have to be on duty all day and work very hard in the greenhouses due to management inefficiencies.

Wireless sensor networks are a modern technology which integrates the knowledge of sensors, automation control, digital network transmission, information storage, and information processing. Currently wireless sensor network technology has been mostly applied to environmental monitoring. In this paper, a vegetable greenhouse architecture is proposed to achieve scientific cultivation and lower management costs in the aspect of environmental monitoring. According to the analysis of the features of greenhouse environment, a practical and low-cost greenhouse monitoring system is designed based on wireless sensor network technology in order to monitor key environmental parameters such as the temperature, humidity, and soil moisture [1–6].

## 2. System Architecture

### 2.1. System Requirements Analysis

Each greenhouse is equipped with one wireless sensor network node, and the node is connected to the temperature, humidity and soil moisture sensors to measure their values inside and outside greenhouses, respectively. An antenna stretches out of each greenhouse to collect data on a predefined interval, meanwhile an *ad-hoc* wireless network is built. Consequently data are transmitted to a base station, where they are packed and sent to the data center in Beijing on a predefined schedule in order to achieve real-time data release in the WEB. The base station is placed where farmers can easily access real-time monitoring data [7].

This system can achieve the following functions: (1) automatic collection of monitoring data for all greenhouses; (2) periodical transmission of the monitoring data and any alarm messages through matching the greenhouse ID to the greenhouse owner’s phone number; (3) rolling and displaying the information on the screen of the base station; (4) acquisition of the monitoring data of the specified greenhouse with text messages being sent by the manager; (5) sending of the real-time greenhouse monitoring data to the Beijing data center via the GPRS network.

### 2.2. System Architecture Design

**Figure 1.**
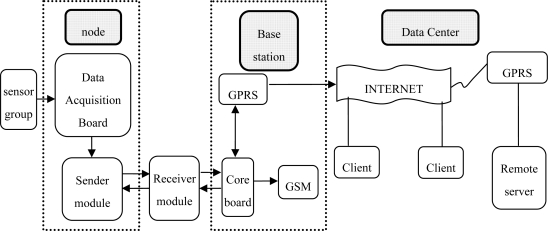
Architecture of greenhouse monitoring system based on wireless sensor network.

The system consists of three modules, which are a node module, a base station module and a data distribution module (Figure 1). The node module is placed inside greenhouses, and the base station module is placed in public areas outside the greenhouses. The base station is equipped with a LCD screen so that the real-time values of temperature and humidity, both inside and outside greenhouses, and soil moisture can be observed.

**Figure 2.**
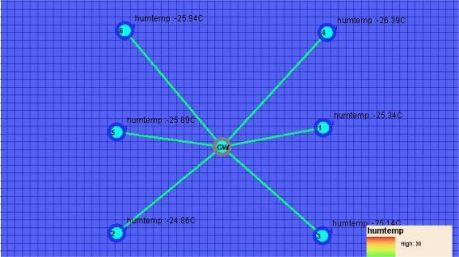
Topological structure of the system.

The relationship between the nodes and the base station is illustrated by a star topology structure as shown in Figure 2.

The WEB releasing module is installed in the data center in Beijing; in fact, it can be installed in any computer with a fixed IP. The system adopts two network communication modes: (1) a wireless network formed between the nodes and the base station through the 802.15.4 protocol; (2) a GPRS network between the GPRS transmission module in the base station and the GPRS transmitter module at the WEB releasing module.

## 3. System Functional Modules

### 3.1. Embedded Operating System ZKOS

**Figure 3.**
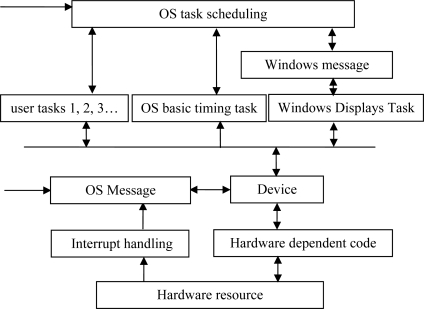
Architecture of ZKOS.

The proprietary embedded operating system ZKOS (Shingle Operation System) has a small amount of code, and is less dependent on system hardware features such as stacks, registers, timers and interrupters. Therefore, it can be implemented on different types of mono-chips [8]. The architecture of embedded ZKOS operating system is shown in Figure 3.

ZKOS is a preemptive, priority-based, real-time, and multitasking operating system kernel, in which the high priority tasks with displace low priority tasks based on CPU privilege; the priorities of each task are different, therefore, the operation sequence of the tasks are arranged by the system according to these priorities. The real-time features of the tasks with high priority are thus ensured, and the scalability of the functions is improved by the multi-tasking feature. Additionally, ZKOS also features protection of shared resources.

### 3.2. Sensor Node Module Hardware and Software Design

**Figure 4.**
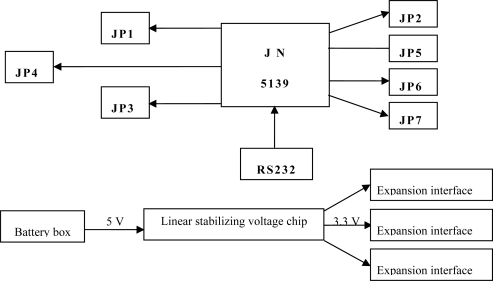
System architecture of the node module.

The sensor node module consists of the CPU, the 2.4G wireless transceiver module, and the data acquisition channel (Figure 4).

The 2.4G wireless transceiver module and the central processing unit (CPU) are integrated into JN5139 for data exchange among the base stations, thus an internal wireless network is built. In order to access various types of sensors the data acquisition channel provides a variety of signal interfaces, including: (1) a standard, multi-channel, 4–20 mA, analog signal interface (e.g., J1-J2); (2) standard interfaces, such as 232 interface, 485 interface and SPI interface; (3) customizable serial I/O interface; (4) interfaces that facilitate the expansion of power supply [9].

As the 2.4G wireless transceiver module receives the signal from the base station, the awaiting sensor set start to gather the temperature and humidity data, and transmit the data digitally to the CPU in JN 5139. The CPU will pack the data, and transmit the data to the base station through the 2.4 G wireless transceiver module. All transactions are classified into four tasks by the operating system embedded in the CPU:

Task 1. Schedule the procedure of A/D conversion for all analog channels (>10 us, may be manually specified), and convert the digital value to the real value.
Task 2. Schedule the communication procedure for all data channels (>0.1 ms, may be manually specified), and get the sample data from sensors.
Task 3. Schedule the data packing procedure (>1 min, may be manually specified), and send the packet to the base station by the 2.4G wireless transceiver module.
Task 4. Reset the ON/OFF status and the sampling interval for each sensor according to the message updated by the base station. Once a task is accomplished, the node becomes dormant.

#### 3.2.1. Design of the Interface with the Sensor

**Figure 5.**
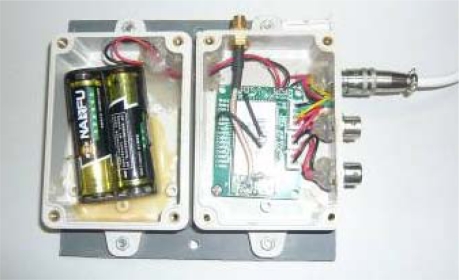
An encapsulated node.

Considering the real environment, the circuit board of node is tailored, and the interfaces compatible with a potentially hostile environment are selected. The battery set and the circuit board are encapsulated in separate packages, making it convenient for battery replacement and circuit protection (Figure 5).

#### 3.2.2. Design of Node Power Management System

**Figure 6.**
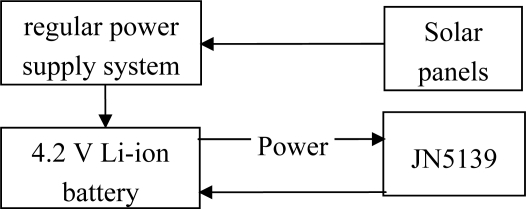
The composition of the node power management system.

The node power management system consists of some solar panels, a 4.2 V 2 AH Li-ion battery and a regulated power supply system (Figure 6). The power management system can provide a lasting and stable power supply for the system; it can regularly test the battery, and shut down the system or turn off the charging device in case of too-low or too-high power.

#### 3.2.3. Software Implementation of Node Modules

##### (1) Data Format

Before they are sent to the base station, the data are collected through nodes in certain format, which is shown as Table 1.

##### (2) Node Working Process

Once a node module is connected to the supply, first the battery voltage is checked. Any voltage under 3.5 V is considered insufficient. To ensure that the battery will not malfunction due to over discharge, the system will set the next starting time as 2 hours later, and immediately enter a dormant state; when the battery voltage is normal, the node will send networking information to the base station.

To reduce the communication conflicts resulting from nodes simultaneously sending networking information, the nodes will delay sending the information based on their own serial numbers (such as Node 1 sending the network information after 5 ms, Node 2 sending information after 10 ms, *etc*.). If a node does not receive the confirmed information from the base station by sending network information within 500 ms, the system will judge the networking times; once it is exceeded 5 times, the system will stop networking and directly go into a state of hibernation.

**Figure 7.**
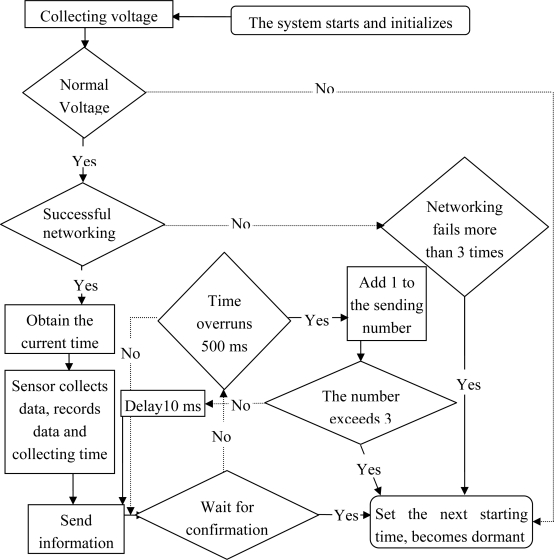
Nodes flow chart.

If the node network is successfully built, the current time and the parameters are obtained from the confirmed information sent through the base station networking to update the node configuration information and the current time; then it starts sampling the sensors and recording the sampling time; the node packs the sampling data, transfers it to the station and waits for its confirmation; if it does not receive the confirmed information from the base station by sending the data within 500 ms, which means the data delivery failed, and if it fails to send data after three tries, then the network breaks down and the system will go into hibernation (Figure 7).

### 3.3. Base Station Module Hardware and Software Design

**Figure 8.**
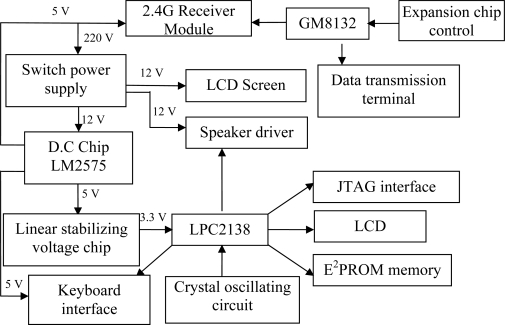
The architecture of base station module.

The base station module is the core component of the whole system, and consists of a CPU, a GPRS communication module, the 2.4G wireless transceiver module, and a LCD display (Figure 8). Transactions are managed by the base station module through the ZKOS operating system. The GPRS remote communication module is designed with the GPRS/GSM integrated package whose power consumption is only 4 Watts, making it more suitable for outdoor applications.

**Figure 9.**
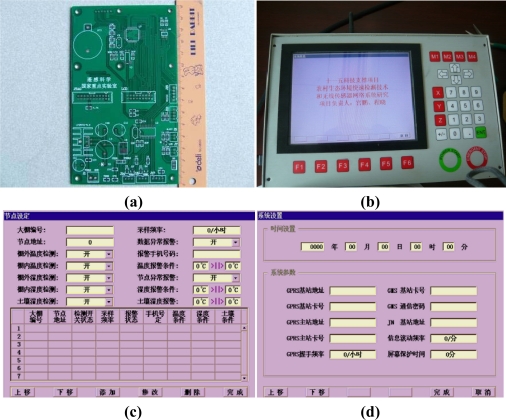
Control configuration page of base station. **(a)** mainboard of base station; **(b)** the base-station system with LCD display, keyboard and wireless transceiver module; **(c)** interface of node configuration; **(d)** interface of system configuration.

ZKOS contains a timer that periodically sends a command through a serial port to the 2.4G wireless transceiver module to transmit current temperature and humidity. After the command is sent, ZKOS is kept in the awaiting status. When it gets current temperature and humidity, the LCD display is refreshed. According to the specifications of the TCP/IP protocol of ZKOS, the data will be packed, processed, and sent to the GRPS module after a predefined interval so that the specified host can obtain the data through the China Mobile network. ZKOS also supports the reverse operation, which means the packet can be unpacked and sent to the WEB distribution module. The operation can be configured by the keyboard in the base station, such as manually setting sampling intervals, GPRS transmission intervals, ID of GPRS, threshold of alarm values for temperature and humidity, mobile phone number which is used to receive alarm messages and the status of LCD screen, *etc*. (Figure 9).

ZKOS is embedded in the CPU and controls the related hardware to accomplish related operations; all transactions can be classified into four tasks:

Task 1. Schedule the procedure of A/D conversion for all analog channels (>10 us, may be manually specified), and convert the digital value to the real value.
Task 2. Schedule the communication procedure for all data channels (>0.1 ms, may be manually specified), and obtain the sample data from the sensors.
Task 3. Schedule the data packing procedure (>1 min, may be manually specified), and send the packet to remote INTERNET server through the GPRS module.
Task 4. Reset the operation status of system, or the relevant execution according to the parsed command received by the GSM module.

#### 3.3.1. Design for the JN5139 Module

**Figure 10.**
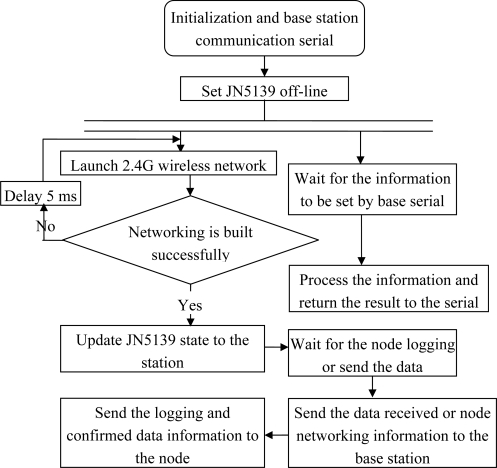
The flow chart of the 2.4G (JN5139) program in the base station.

When JN5139 is connected to the power supply for the initialization, it starts building the network, and waits for its successful communication with the base station or nodes. After the node logging data are received, the confirmation will be sent back and the latest base station time and operating parameters of the node are added. This node is then added into its own node management list, while the node on-line information is uploaded to ARMLPC2138 in the base station. When JN5139 receives uploaded data through the node, it will transmit the data to the base station ARM. The confirmed information is also sent back to the node; when JN5139 receives any information from the base station, it will save the data and respond to the base station (Figure 10).

#### 3.3.2. Design for the GPRS Module

After initialization, the system enters the serial communication state and the power of GPRS module is turned on. The GPRS module boots, waits for its initialization, lands the mobile phone network, and then sets the operating parameters of the GPRS module.

The TCP/IP connection with the remote server is conducted; if the connection fails a couple of times, it will automatically turn off the module and repeat the above steps; when the connection is fulfilled, the networking maintaining communication is repeated every five minutes (if there is no mission data sent during this period).

Data is waiting to be uploaded through the GPRS module. If it is text messages, then the message content will be analyzed and whether the message format and password are correct is determined. If correct, the execution results will be sent back to the cell phone in the form of text messages, and the read message will be deleted from the SIM card in case that the SIM card is full and cannot receive new text messages.

When an alarm phone number is set in the system, and at the same time the data uploaded in the node meet the alarming conditions, the text messages in the specified format will be sent to the designated phone number.

**Figure 11.**
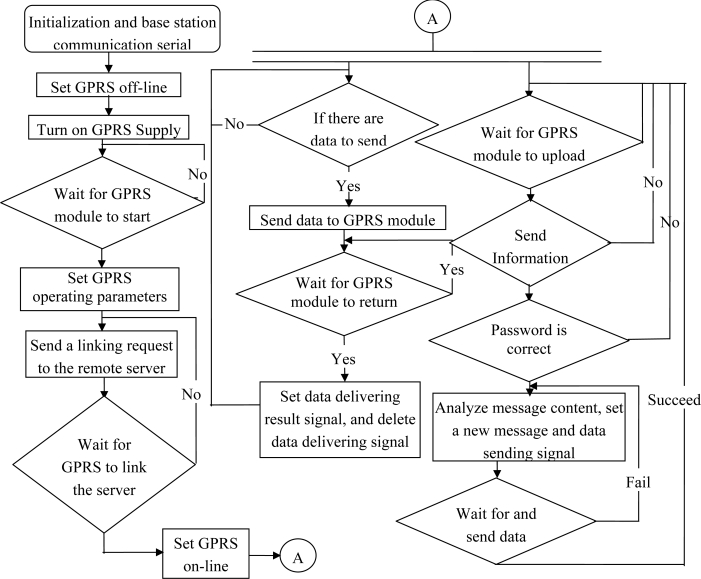
The base station and the GPRS communication flow.

Once per second, the module signal strength from the GPRS module is read and displayed on the screen (Figure 11).

#### 3.3.3. Interfacing Implementation of LPC2138 and JN5139

**Figure 12.**
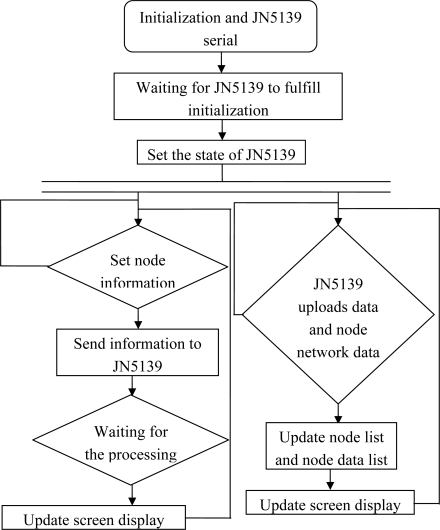
Interfacing implementation of LPC2138 and JN5139 flow.

Since the base station module is connected to the power supply, the JN5139 module is initialized and its working state is set; then it is followed by implementation of the two parallel tasks on the JN5139 (Figure 12).

##### (1) Parameter Setting Tasks

The base station waits for the user to set the node information. The information will be sent to JN5139 module once it is obtained. Then the station waits for the JN5139 module to return information of the processing result, and updates the screen accordingly. This task will be implemented by a loop.

##### (2) Node Uploading Data Processing Tasks

At this time, the base station waits for JN5139 to upload the node networking data. Then the updated data are stored in the node information list and the data collection list of the base station. After that the information is renewed on the screen. This task will be implemented by a loop.

#### 3.3.4. Implementation of Data Processing Tasks

**Figure 13.**
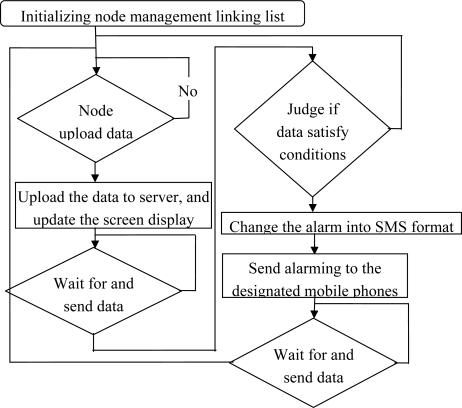
Implementation of data processing tasks flow.

The base station module initializes the node management list prior to the implementation of the task, and then waits for the node to upload sample data; the base station sends the correct sampled data to the server via GPRS, while the data are displayed on the screen of the base station; then it is determined whether the sampled data exceeds the proposed alarm conditions, if an alarm condition is satisfied, the base station will send alarm information via GSM SMS to the designated mobile phones; and the data are expected to be uploaded by the next node. This task will be implemented by a loop (Figure 13).

#### 3.3.5. Other Tasks

##### (1) Message Loss Handling

To avoid losing messages, the system establishes message numbering and a response message. All the data messages correspond to a specified response message; when a node is sending messages, it must receive its response message, thus it can be considered as a successful communication; each message has a message numbering; when communication is finished, 1 will be automatically added to the message numbering; when the response is not received within a fixed time interval or an error prompts into the response message, the message will be re-transmitted; if the received message numbering is greater than the previous one, this message will be identified as a valid message processing; if it is determined to be an invalid one, it should send the corresponding response message in order to inform the sending terminal on whether the current communication is normal.

##### (2) Delay Processing

In the network communication, it may occur that multiple nodes simultaneously send data and block the communications. In this study, the nodes will adopt different time delays, so that the above situation can be solved. To ensure a different time delay assigned for each node, the delay time of each node is associated with the node number. The higher priority the response message has, the shorter the time delay is.

##### (3) Time Synchronization of Data Collecting by Nodes

To ensure accuracy and consistency in the data collection time of nodes, a precise time source is used in the system for calibrating the time of the various network nodes. Because a star network is used and there are no repeaters in the system, the time for the communication from the base station to the nodes is very short, but relatively stable and fixed. The system is connected to a GPS, using GPS satellite timing to calibrate the time of the base station. Then the time of nodes is corrected through the base station when the node is online for the first time. This ensures that the error between the various nodes and the error between the node time and real time are kept within the ms range.

### 3.4. Implementation of Software and Hardware Watchdog

The 2.4G wireless network is severely influenced by the outside environment, and may be unstable. To ensure the system work normally, software and hardware watchdogs are designed.

**Figure 14.**
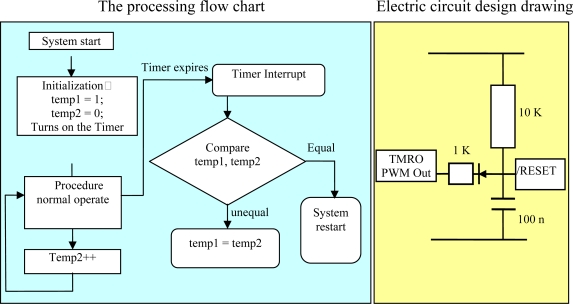
Flow chart of software watch-dog system, and circuit design of hardware watch-dog.

1. Software watchdog: when the CPU timer works normally, but the program works abnormally, the software watchdog converts the operation into restart mode by the timer, so that the system can restart automatically.
2. Hardware watchdog: as an individual component, the timer works in a way of interruption, when the operation of main program goes wrong, the interrupted program can still operate normally to guarantee that the timer can work normally (Figure 14).

## 4. Data Release Module

**Figure 15.**
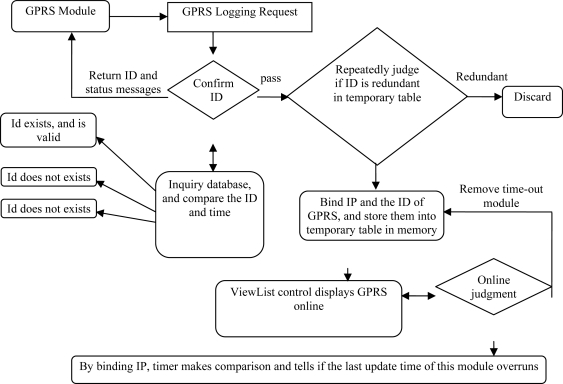
TCPServer workflow.

The data release module is installed in the Beijing host server, comprising the TCPServer program and the WEB release program. The TCPServer is used to receive and analyze data from the remote GPRS communication module. This function is achieved by monitoring a specified port and receiving the real-time transmission data with a multithreading technique and the TCP/IP communication protocol. The operation mode is a fussy 3-layer architecture including surface layer, logical layer, and data layer (Figure 15).

**Figure 16.**
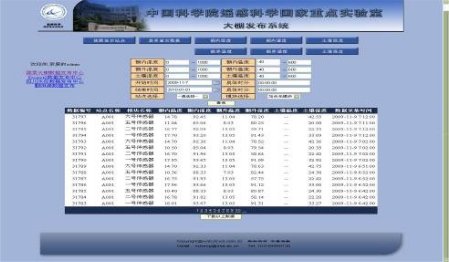
WEB Release System Screenshots.

The implementation of WEB release is achieved by the combination of the newest Microsoft’s MVC framework with the factory mode and the 3-layer architecture, which has both foreground and background functions. The foreground functions include logging in, data display, making inquires, and *etc*. (Figure 16).

The users are classified into three levels as administrators, members, and visitors. The administrators have the highest priority, and can manipulate all data; the next are the members who can download data only after logging into their accounts; the visitors (default level) has the lowest priority, and can only enter the system and browse the data but with no permission to download it.

The data in the database is displayed in a data table in a reversed order on each page. The page is refreshed at specified intervals. Users can select stations and nodes by drop-down menu, and look up all data within a certain period in database. The background functions mainly include adding, deleting, checking, and modifying the information of sites, nodes and users; the backstage program is implemented on WEB, so the administrators can log into the system with their accounts and operate the system remotely.

## 5. Field Test of Vegetable Greenhouse Monitoring System

Since 2008, an experimental wireless-sensor-network-based monitoring system for vegetable greenhouses has been running in the Ansai Hou Trench Gate Village demostration area. Since 2009, the long-term field-test has been carried out in the Olympic Games Science and Technology Park of Chinese Academy of Sciences, and satisfactory experimental data have been observed.

With regards to the aspect of energy consumption, two No. 5 Nanfu shaped ring cells can work for more than six months under a 10-minute sampling frequency condition. As for the transmission distance and the ability to cross barriers, even if the base station is placed on 10-story towers, the communication distance can reach 2,000 m under barrier-free circumstances, and if there is a three-reinforced-concrete-tower barrier, the longest stable transmission distance can approach 852 m.

Regardless of all kinds of human factors, the system can work normally for six consecutive months without maintenance. The bit error rate is found at ±2% upon the analysis of the returned historical data.

In the installation and operation procedure the following features were observed:

1. The quality of the 2.4G network is significantly affected by environmental factors, which is attributed to the reflection and absorption of electromagnetic waves by the surrounding environment. The greenhouses in the Ansai Hou Trench Gate Village demonstration area are built of the local loess; if the node and the antenna are both placed inside the shed, the network cannot be connected. It is found during the experiment that the loess could effectively absorb the electromagnetic wave. However, the experiments in the Olympic Games Science and Technology Park of the Chinese Academy indicate that two nodes in the network can still be connected even at a distance of 1.2 km with four buildings between them.
2. The 2.4G wireless network and Wi-Fi network will interfere with each other; because the Zigbee-based 2.4G wireless network and Wi-Fi network both operate within the ISM frequency band, interference is likely to occur.
3. The height of the node’s antenna that connects to the 2.4G transmitter module cannot be higher than that of the base station antenna which is connected to the 2.4G receiver module. The whole system adopts omni-directional antenna. In the test, if the receiver’s (base station) antenna height is lower than that of the transmitter (node), the signal will be poor and the transmission distance will be constrained [10].

## 6. Conclusions

Developed on an independently-developed wireless sensor network platform, the reported monitoring system for vegetable greenhouses is a successful combination of wireless sensor network technology and the mobile communication technology in digital agriculture [4–5]. This system possesses the following merits: (1) it is low-cost, scalable and reliable with good processing capability; (2) the design of hardware and software watchdogs can ensure the system will be online in a real-time manner; (3) multiple interface designs allow the system to access multiple sensors; (4) through the Internet users can make inquiries of the real-time environmental parameters inside the greenhouse, and can control the facilities in greenhouses by making use of China Mobile’s SMS remotely.

Our aim of the monitoring system for vegetable greenhouses proposes higher requirements for the system, such as stability and fast reaction. As the field conditions change frequently, improvements to the system are needed. Particularly, the capability to deal with abnormal situations needs to be strengthened.

## Figures and Tables

**Table 1.** node sent to the base station data format.

**Name**	**Length (Byte)**	**Meaning**
Message header	1	Regular byte 0XD7
Serial number of nodes	1	The serial number of nodes (set by users)
Message numbering	1	It’s used to identify message currently delivered
CRC check code	2	Data CRC check code
Data length	1	Data length (excluding the first three items and its own length)
Sensor state	1	Whether the sensors are valid
Sensor data	N	The data of sensors are concerned about temperature, humidity and battery voltage
Sampling time	6	The time when data are sampled
